# Feasibility Study of a Novel Post-Processing Method Based on Microscale Laser Shock Peening Without Coating for Selective Laser Melting-Fabricated Components

**DOI:** 10.3390/ma19204376

**Published:** 2026-10-18

**Authors:** Congkai Shi, Xiangfan Nie, Song Shu, Rubin Cui, Hongbing Li, Shixi Li, Peiwen Xu

**Affiliations:** 1 National Key Laboratory of Aerospace Power System and Plasma Technology, Air Force Engineering University, Xi’an 710038, China; 2 National Key Laboratory of Aerospace Power System and Plasma Technology, Xi’an Jiaotong University, Xi’an 710049, China; 13772133805@163.com (R.C.);; 3 State-Owned Machinery Factory in Wuhu, Wuhu 241007, China; 4 School of Aeronautics, Chongqing Jiaotong University, Chongqing 400074, China

**Keywords:** selective laser melting, microscale laser shock peening without coating, micro-melting effect, severe plastic deformation, surface integrity, fatigue performance

## Abstract

**Highlights:**

• μLSPwC is a direct surface post-processing method for locally complex SLM surfaces.
• Surface roughness decreases over 20% across 17-4PH, TC4, and GH4169 alloys.
• μLSPwC induces deep compressive stresses and hardening layers exceeding 300 micrometers.
• Rotating bending fatigue life increases to up to 247.9 times that of the untreated condition (TC4).

**Abstract:**

Selective Laser Melting (SLM) has been widely employed for fabricating lightweight and integrated components for aircraft and aero-engine applications. However, SLM-fabricated components, especially those containing locally complex surface regions, often suffer from burrs, balling, microcracks, and tensile residual stress (TRS). These issues are difficult to eliminate by conventional machining or heat treatment (HT) alone. Consequently, such defects degrade fatigue performance and restrict the practical engineering application of SLM-fabricated components. This study proposed a novel direct surface post-processing method utilizing microscale laser shock peening without coating (μLSPwC) for locally complex surface regions of SLM-fabricated components. The process was designed to combine laser-induced local micro-melting for surface defect mitigation with shock-induced plastic deformation for near-surface strengthening. SLM-fabricated 17-4PH (AISI 630, UNS S17400) stainless steel, TC4 (Ti-6Al-4V) titanium alloy, and GH4169 (Inconel 718, UNS N07718) nickel-based superalloy were treated to assess the impact of μLSPwC on surface morphology, roughness, residual stress, microhardness, and rotating bending fatigue life. The surface roughness of all three materials dropped more than 20% after treatment. Furthermore, μLSPwC introduced compressive residual stress (CRS) layers and hardened layers exceeding 300 μm. The rotating bending fatigue life was improved by more than 20 times, and in some cases by over two orders of magnitude, at the selected stress levels. Therefore, μLSPwC is a promising post-processing technique for locally complex surface regions on SLM-fabricated components, particularly in situations where hot isostatic pressing, HT, or conventional surface machining is impractical or ineffective.

## 1. Introduction

Selective laser melting (SLM) is a primary additive manufacturing technology for metallic materials and has been extensively employed in the fabrication of lightweight and geometrically complex components for aircraft and aero-engine applications [1,2,3,4]. Although SLM provides high dimensional accuracy and excellent design flexibility, the fabricated components often exhibit compromised surface integrity (burrs, balling, microcracks) and tensile residual stress (TRS) [5,6]. Such factors are especially harmful to fatigue performance, since they intensify local stress concentration and accelerate early crack initiation.

To ensure the reliability of SLM-fabricated components, post-processing methods, for instance, hot isostatic pressing and stress-relief annealing are often required [7,8,9]. However, these conventional methods face practical limitations when applied to components with locally complex surface features. In confined regions, conventional machining is often unfeasible, and heat treatment (HT) alone fails to eliminate surface defects or restore the near-surface stress state [7,9].

Mechanical surface modification techniques, including shot peening (SP), rolling, and low plasticity burnishing (LPB), have been extensively used for surface integrity enhancement of additively manufactured metallic materials [10,11,12,13]. While SP can enhance fatigue performance through the introduction of compressive residual stress (CRS) and the alleviation of stress concentration [14,15,16,17], rolling focuses on refining the near-surface microstructure to enhance local mechanical properties [18,19,20]. LPB is particularly attractive because it produces comparatively deep CRS fields while causing only limited cold work [12]. Nevertheless, for these mechanical methods, direct removal of surface balling, burrs, and microcracks remains rather restricted, especially in locally complex regions [21].

Laser shock peening (LSP) generates high-pressure shock waves via pulsed laser irradiation, which introduce deep CRS and promote beneficial microstructural changes [22,23,24]. Unlike traditional mechanical treatments, LSP offers superior geometric flexibility, enabling treatment of localized areas. When the absorbing coating is omitted under controlled conditions, direct laser–surface interaction may locally melt asperities and loosely bonded particles. This may enable simultaneous improvement in surface morphology and near-surface mechanical properties.

This study proposes a microscale laser shock peening without coating (μLSPwC) process as a direct surface post-processing route for SLM-fabricated components. The influence of this process on surface integrity was systematically investigated by evaluating morphology, roughness, residual stress, and microhardness. Furthermore, its feasibility for enhancing fatigue performance was experimentally validated through rotating bending fatigue tests at the selected stress levels.

## 2. Materials and Methods

The SLM system employed was an EP-260 machine (Eplus3D Additive Technology Co., Ltd., Hangzhou, China). Processing was performed under a high-purity nitrogen atmosphere, with the oxygen content maintained below 1% throughout fabrication. Three representative aerospace alloys—17-4PH stainless steel (AISI 630, UNS S17400), TC4 (Ti-6Al-4V) titanium alloy, and GH4169 (Inconel 718, UNS N07718) nickel-based superalloy—were selected to assess the effectiveness of μLSPwC on different alloy systems. Table 1 presents the chemical compositions of the powders. The SLM fabrication employed a 1070 nm laser with a 90 μm spot diameter. Processing conditions were fixed at 200 W power, 1000 mm/s scanning velocity, and 0.1 mm hatch spacing. Build layer thickness varied by material, using 40 μm for 17-4PH and GH4169, and 30 μm for TC4.

**Figure 1:**
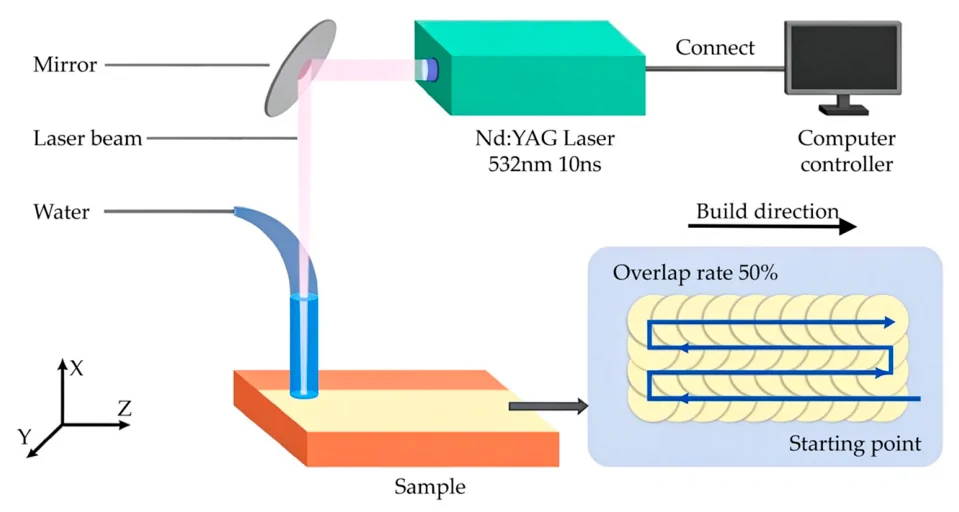
Schematic of coaxial water–laser μLSPwC for SLM-fabricated specimens.

While conventional LSP utilizes high-intensity laser pulses to induce GPa-level shock waves, thereby driving plastic deformation and establishing CRS fields in the near-surface region [25,26], the absorbing coating was omitted in this study to facilitate direct laser-material interaction. This configuration facilitated localized micro-melting of surface burrs and balling while still generating sufficient shock pressure to induce near-surface plastic deformation. To avoid excessive ablation and thermally induced damage, the pulse energy was controlled at the millijoule level, and the beam was focused to a microscale spot size to maintain the laser power density in the GW/cm^2^ range. A coaxial water–laser delivery configuration (Figure 1) ensured a stable water confinement layer. Table 2 summarizes the detailed treatment parameters. Hereafter, specimens not subjected to μLSPwC are referred to as unLSP, whereas treated specimens are denoted as μLSPwC.

The surface morphologies of the SLM-fabricated specimens before and after μLSPwC treatment were observed with a ZEISS EVO-10 SEM (Carl Zeiss Microscopy GmbH, Oberkochen, Germany). Surface profiles and roughness were quantified using a KC-X1000 laser confocal microscope (Nanjing Kathmatic Technology Co., Ltd., Nanjing, China). For each condition, measurements were performed on at least three specimens, with a cut-off length of 10 mm. The balling density was quantified using ImageJ (NIH, version 1.53e). At least three SEM images per condition were analyzed. Spherical particles were segmented using the auto-threshold function and filtered by circularity to isolate near-spherical features. Balling density was defined as the number of particles per unit area, with identical selection criteria applied to 17-4PH, TC4, and GH4169.

Surface and depth-resolved residual stress measurements were conducted using a PROTO LXRD-MG2000 system (Proto Manufacturing Ltd., LaSalle, ON, Canada) based on the sin^2^Ψ method, with a measurement accuracy of ±20 MPa. Different targets were selected to match the crystallographic planes of interest for each alloy: Cr Kα radiation on the {211} plane of 17-4PH at 2θ ≈ 156.1°, Cu Kα on the {213} plane of TC4 at 2θ ≈ 142°, and Mn Kα on the {311} plane of GH4169 at 2θ ≈ 152°. Measurements were performed using 9 ψ angles, with an irradiated area of approximately 2 mm^2^. Depth-resolved residual stress measurements were obtained through sequential electrolytic polishing in an electrolyte consisting of 90% methanol and 10% perchloric acid.

Surface and subsurface microhardness were measured using a 200HV-5 microhardness tester (Zhengzhou Huayin Test Instrument Co., Ltd., Zhengzhou, China). A load of 200 gf with a dwell time of 15 s was applied for 17-4PH and GH4169, whereas a load of 100 gf with the same dwell time was used for TC4. At each depth, three indentations were made at different locations and averaged. The spacing between adjacent indents was maintained at more than three times the diagonal length of the indentation to avoid interaction between neighbouring plastic zones. Cross-sectional specimens were prepared by cutting, mounting, and polishing to a mirror finish.

EBSD characterization was performed using a Thermo Scientific Nova NanoSEM 450 (Thermo Fisher Scientific, Waltham, MA, USA) equipped with an EDAX Hikari Plus detector (EDAX Inc., Mahwah, NJ, USA). The acceleration voltage was 20 kV. Maps were acquired over the near-surface region with a step size of 0.5 μm.

**Figure 2:**
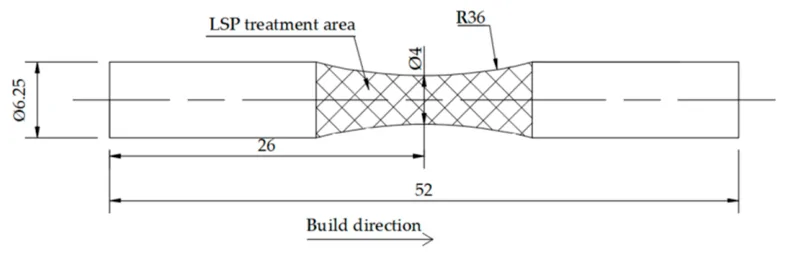
Schematic of SLM-fabricated rotating bending fatigue specimen.

High-cycle fatigue tests were conducted using a QBWP-10000 rotating bending fatigue testing machine (Changchun Qianbang Testing Equipment Co., Ltd., Changchun, China). For all three alloys, the tests were performed at room temperature according to the Chinese aviation industry standard HB 5152-1996, “Metallic Materials—Rotating Bending Fatigue Test Method at Room Temperature”, with a stress ratio of R = −1 and a rotational speed of 3000 rpm [27]. The applied stress levels were 400 MPa for 17-4PH, 315 MPa for TC4, and 400 MPa for GH4169, corresponding to approximately 30% of the ultimate tensile strength of the respective materials. The specimen geometry is shown in Figure 2. Three replicate specimens were tested for each material in each condition (i.e., unLSP and μLSPwC). All fatigue tests were continued until specimen fracture; no run-outs were observed for any specimen within the tested stress levels.

## 3. Results and Discussion

### 3.1. Surface Topography and Roughness

**Figure 3:**
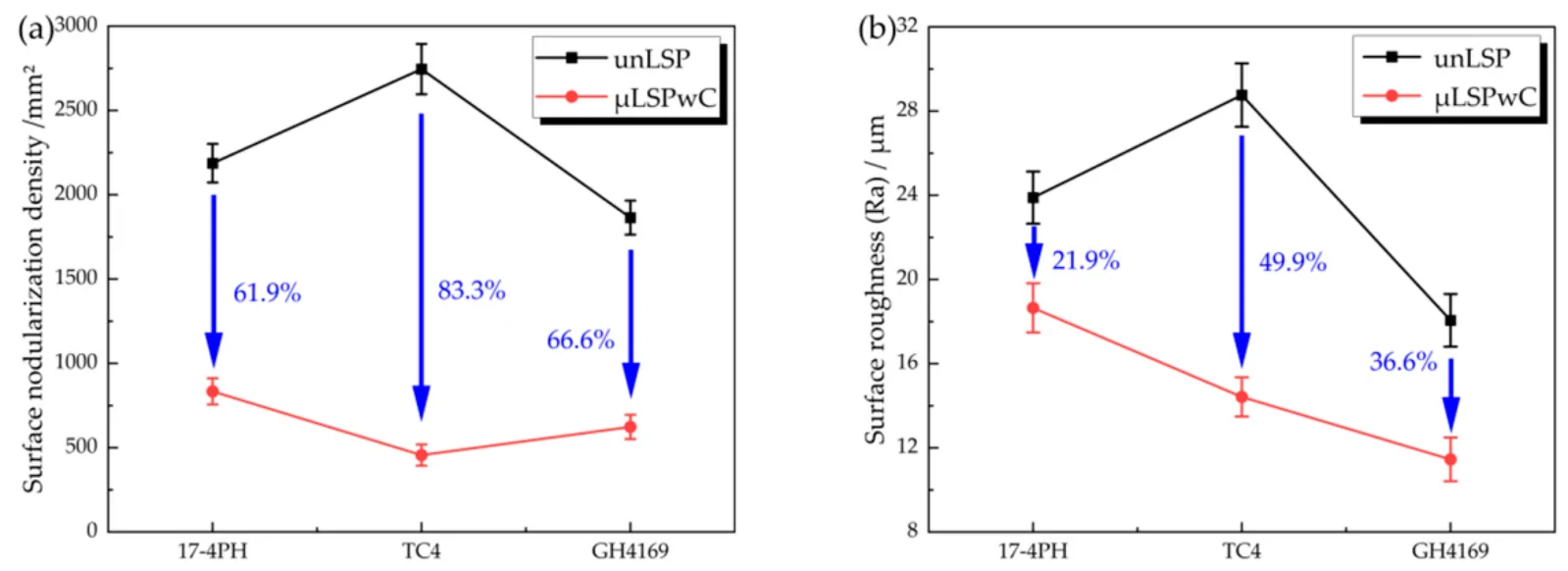
Effect of μLSPwC on surface quality: (**a**) surface balling density and (**b**) surface roughness (Ra) of 17-4PH, TC4, and GH4169 before (unLSP) and after μLSPwC treatment. The blue arrows and associated percentage values indicate the reduction of μLSPwC relative to unLSP by 61.9%, 83.3%, and 66.6% for balling density, and by 21.9%, 49.9%, and 36.6% for Ra, corresponding to 17-4PH, TC4, and GH4169, respectively.

In the unLSP condition, all SLM surfaces exhibited numerous adhered particles, along with evident burrs and balling. Following μLSPwC treatment, the surface balling density decreased markedly in all three alloys. As quantified in Figure 3a, the balling density decreased from 2187 to 834 mm^−2^ in 17-4PH, from 2745 to 456 mm^−2^ in TC4, and from 1864 to 623 mm^−2^ in GH4169, corresponding to reductions of 61.9%, 83.3%, and 66.6%, respectively. The effect of μLSPwC on surface roughness (Ra) is shown in Figure 3b. Following treatment, Ra decreased from 23.89 to 18.65 μm for 17-4PH, from 28.76 to 14.42 μm for TC4, and from 18.06 to 11.45 μm for GH4169, corresponding to reductions of 21.9%, 49.9%, and 36.6%, respectively. TC4 demonstrated the most significant improvement, exhibiting the highest reduction rates in both balling density and surface roughness among the three alloys.

**Figure 4:**
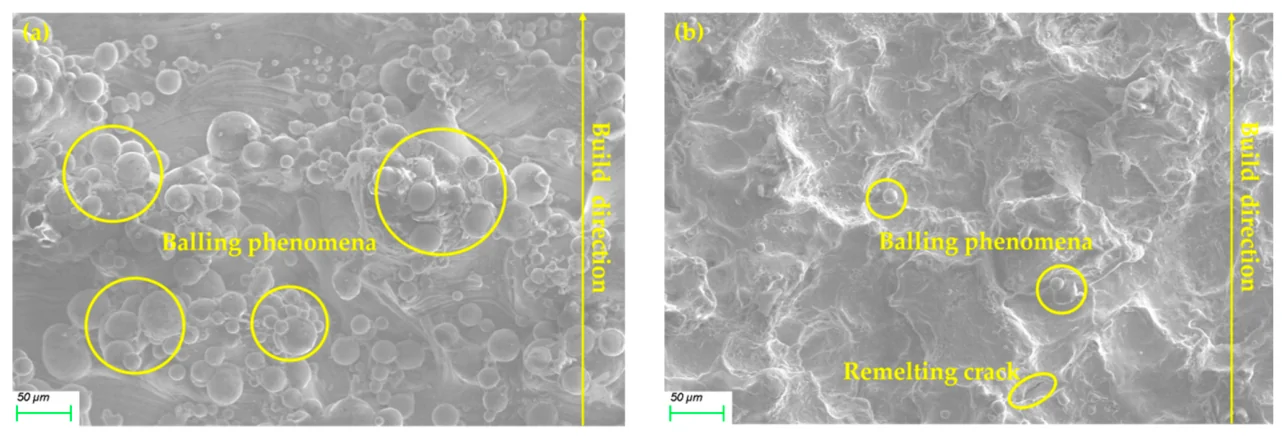
Typical surface morphology of SLM-fabricated TC4 before and after μLSPwC treatment: (**a**) unLSP; (**b**) after μLSPwC treatment.

Representative SEM morphologies of TC4 (Figure 4) revealed surface modification induced by μLSPwC. The direct laser-surface interaction is a thermomechanical process in which localized thermal effects can partially melt surface burrs and balling particles, followed by rapid resolidification [28]. Although a small number of remelting cracks suggest localized thermal damage, the process effectively removes or reflows these defects, forming smoother surface regions and substantially reducing roughness. It should be noted that, without an absorption coating, ablation, vaporization, and recast-layer formation can accompany shock-wave-driven plastic deformation [29], which may also play a role in the observed modification.

### 3.2. Residual Stress and Microhardness

**Figure 5:**
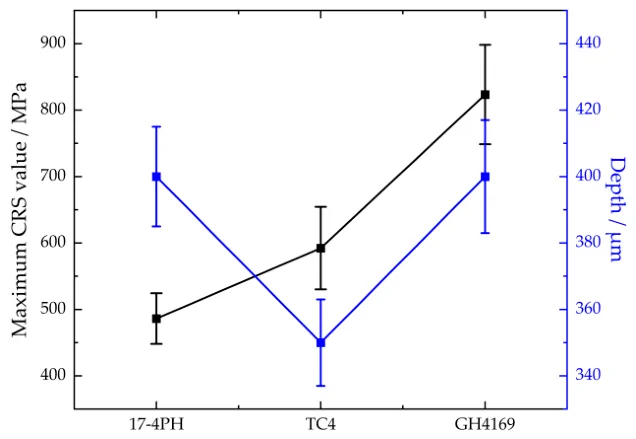
Maximum CRS induced by μLSPwC and its corresponding depth below the treated surface for 17-4PH, TC4, and GH4169. The left axis (black) indicates the maximum CRS value in MPa, and the right axis (blue) indicates the corresponding depth in μm.

Beyond modifying surface morphology, μLSPwC significantly altered the near-surface residual stress state. Figure 5 presents the maximum CRS induced by μLSPwC and their corresponding depths below the treated surface for the three alloys. Maximum CRS values reached 486.2 MPa for 17-4PH (CRS layer ~400 μm deep), 592.3 MPa for TC4 (~350 μm deep), and 823.5 MPa for GH4169 (~400 μm deep). These deep, high-magnitude CRS profiles (exceeding 400 MPa and extending beyond 300 μm) confirm the effectiveness of μLSPwC in imparting subsurface compressive stresses to additively manufactured components. Based on the Fabbro confined-plasma model [30], the peak shock pressure was estimated to be approximately 2–3 GPa; such high pressure levels can induce plastic deformation and compressive residual stresses in metallic materials [31], supporting a significant mechanical shock-wave contribution to the observed compressive residual stresses. However, the mechanical and thermal/ablative effects cannot be quantitatively separated in the present study.

**Figure 6:**
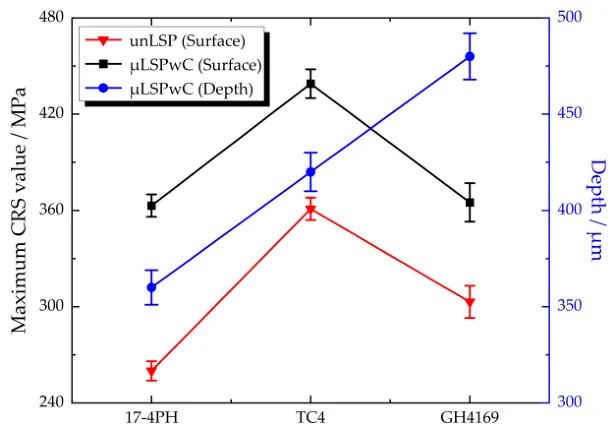
Effect of μLSPwC on microhardness: surface microhardness of 17-4PH, TC4, and GH4169 before (unLSP) and after μLSPwC treatment (left axis), and the corresponding depth of the hardened layer induced by μLSPwC (right axis).

Figure 6 compares the near-surface microhardness distributions before and after μLSPwC treatment. For 17-4PH, surface microhardness increased from approximately 260 HV to 363 HV (a 39.6% increase), and the hardened layer extended to about 360 μm. In TC4, microhardness rose from about 361 HV to 439 HV, corresponding to an increase of 21.6%, with a hardened layer depth of about 420 μm. For GH4169, it increased from about 303 HV to 365 HV, a rise of 20.5%, with a hardened layer depth of about 480 μm. All three alloys exhibited microhardness increases of more than 20% and hardened layer depths exceeding 300 μm after μLSPwC treatment.

**Figure 7:**
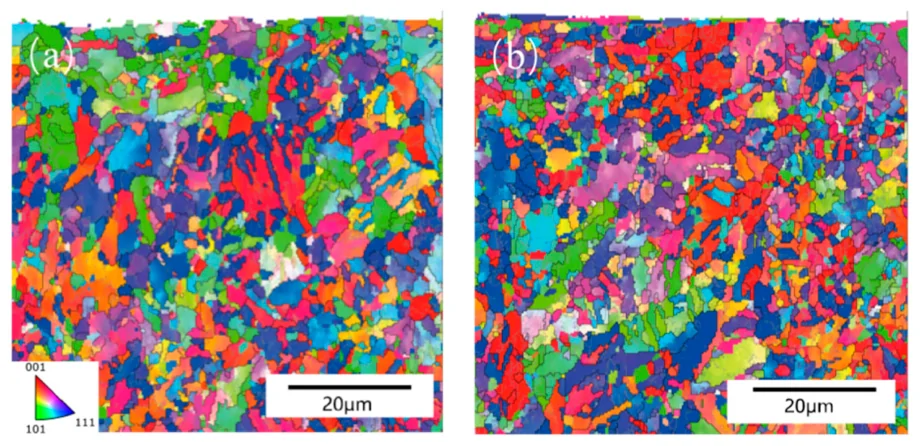
Inverse pole figure maps showing crystallographic orientation of SLM-fabricated 17-4PH (**a**) unLSP (**b**) after μLSPwC treatment. Reproduced from Cui et al. (2026) [33]; Copyright 2026 Elsevier Ltd., Amsterdam, The Netherlands.

The increase in near-surface microhardness can be attributed to severe plastic deformation induced by the laser shock wave. Such deformation is known to generate crystalline defects (e.g., dislocations, deformation twins, and grain boundaries) that contribute to work hardening in metallic materials [32]. As shown in Figure 7 (reproduced from Ref. [33]), the EBSD maps reveal evidence of microstructural modification in the near-surface region of 17-4PH after μLSPwC, consistent with shock-wave-driven plastic deformation.

### 3.3. High-Cycle Fatigue Property

The individual fatigue life data for all tested specimens are summarized in Table 3. As shown in the table, μLSPwC treatment substantially extended the fatigue life for all three alloys compared to the unLSP condition. After treatment, the surface integrity of the SLM-fabricated components was considerably altered: surface defects and roughness were reduced, while deep CRS layers and hardened surface layers were introduced. The fatigue life increased from 34,267 to 963,426 cycles for 17-4PH, from 11,392 to 2,824,308 cycles for TC4, and from 37,125 to 1,303,263 cycles for GH4169, corresponding to 28.1, 247.9, and 35.1 times the untreated fatigue life, respectively. Thus, μLSPwC significantly extended the average fatigue life under the investigated cyclic loading conditions.

**Figure 8:**
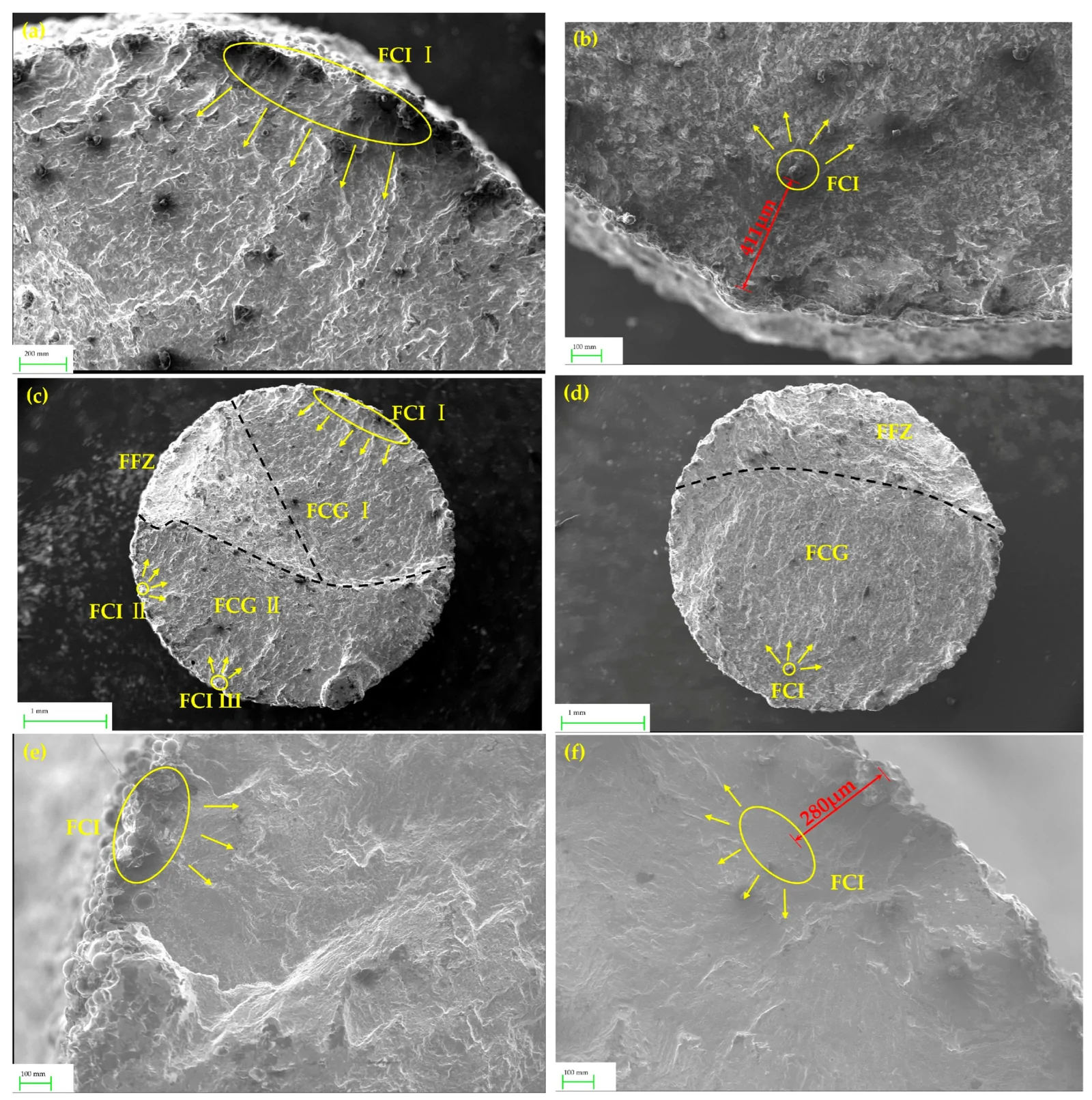
Typical fatigue fracture morphologies before and after μLSPwC treatment: (**a**,**b**) crack initiation sites of GH4169 (unLSP and μLSPwC); (**c**,**d**) crack propagation regions of GH4169 (unLSP and μLSPwC); (**e**,**f**) crack initiation sites of TC4 (unLSP and μLSPwC). The abbreviations denote fatigue crack initiation (FCI), fatigue crack growth (FCG), and final fracture zone (FFZ). The black dashed lines distinguish the FCG and FFZ regions.

Figure 8 presents typical fatigue fracture morphologies of SLM-fabricated GH4169 and TC4 in the unLSP and μLSPwC conditions. Figure 8a,b display that for GH4169, after μLSPwC, fatigue cracks initiated in the subsurface region rather than at the surface, at depths greater than 200 μm below the surface. The reduction in surface defects and roughness markedly lowered the local stress concentration. Meanwhile, the high CRS introduced at the surface partially offset the applied cyclic stress, and the increase in near-surface strength and microhardness suppressed cyclic slip during fatigue loading. The combined effects of these three factors eliminated the conditions necessary for surface crack initiation, therefore suppressing surface fatigue crack initiation. Consequently, cracks initiated in the subsurface region, where the magnitude of CRS and work hardening was lower.

Figure 8c,d demonstrate that after μLSPwC, the crack propagation region of GH4169 exhibited considerable enlargement along with finer striations. This indicates a substantial increase in fatigue crack propagation life and a pronounced reduction in crack growth rate. These effects were primarily attributed to the presence of work-hardened layers and CRS layers with depths exceeding 300 μm, which continuously retarded crack propagation during cyclic loading and thereby significantly extended the fatigue crack propagation life.

For TC4, Figure 8e,f show that μLSPwC shifts the crack initiation site from the surface to a subsurface depth of ~280 μm, consistent with the same suppression mechanism observed for GH4169, although the potential influence of internal defects was not evaluated in this study.

## 4. Conclusions

This study demonstrates microscale laser shock peening without coating (μLSPwC) as a direct post-processing method for SLM-fabricated 17-4PH, TC4, and GH4169. The primary conclusions are summarized as follows:

The surface balling density of SLM-fabricated 17-4PH, TC4, and GH4169 decreased by 61.9%, 83.3%, and 66.6%, respectively, while the surface roughness was reduced by 21.9%, 49.9%, and 36.6%. These reductions suggest that the laser-induced thermomechanical effect likely contributes to the partial melting and reflow of surface burrs and balling particles, leading to a smoother surface morphology. Notably, this surface improvement is achieved without the need for an absorbing coating, which simplifies the process and enables direct treatment of complex geometries.

Maximum CRSs of 486.2 MPa, 592.3 MPa, and 823.5 MPa were introduced into the three materials, with layer depths exceeding 300 μm, indicating that laser shock-induced plastic deformation produces a CRS layer with considerable depth.

The surface microhardness of the three materials increased by 39.6%, 21.6%, and 20.5%, respectively, with hardened layer depths of approximately 360 μm, 420 μm, and 480 μm. This is attributed to severe plastic deformation induced by the laser shock wave, which is known to generate crystalline defects that contribute to work hardening in metallic materials.

The fatigue life of the three materials increased to 28.1, 247.9, and 35.1 times that of the untreated condition, respectively, at the selected stress levels. The improved fatigue life is primarily attributed to reduced surface defects, lower roughness, the introduction of deep CRS layers, and work-hardened surface layers, which collectively suppress fatigue crack initiation and retard crack propagation.

In summary, μLSPwC provides an effective direct post-processing technique for SLM-fabricated components, particularly in locally complex surface regions where conventional machining or heat treatment is difficult to apply effectively.

## Figures and Tables

**Table 1:** Chemical Compositions of the SLM Powders (wt.%).

Material	Element	Content	Element	Content	Element	Content
17-4PH	Cr	16.71	Ni	4.004	Cu	3.164
	Mn	0.562	Mo	0.527	Si	0.344
	Nb	0.156	C	0.029	Fe	Bal.
TC4	Al	5.5	V	3.5	Fe	≤0.25
	C	≤0.08	O	≤0.10	N	≤0.03
	Ti	Bal.				
GH4169	Cr	17–21	Mo	2.8–3.3	Nb	5.00–5.50
	Al	0.3–0.7	Ti	0.75–1.15	C	0.02–0.06
	Co	≤1.0	Ni	Bal.		

**Table 2:** Detailed parameters of μLSPwC treatment.

Material	Wavelength (nm)	Pulse Width (ns)	Frequency (Hz)	Laser Energy (mJ)	Spot Size (μm)	Laser Power Density (GW/cm^2^)	Overlap (%)	Laser Impacts
17-4PH	532	10	500	80	380	7.06	50	1
TC4	532	10	500	76	400	6.05	50	1
GH4169	532	10	500	75	350	7.80	50	1

**Table 3:** Fatigue life of SLM-fabricated 17-4PH, TC4, and GH4169 before and after μLSPwC treatment.

Condition	Specimen 1 (Cycles)	Specimen 2 (Cycles)	Specimen 3 (Cycles)	Average Life (Cycles)	CV (%)
unLSP 17-4PH	30,241	34,967	37,592	34,267	10.87
μLSPwC 17-4PH	1,047,320	874,204	968,755	963,426	9.00
unLSP TC4	11,356	10,481	12,338	11,392	8.16
μLSPwC TC4	2,654,723	2,805,637	3,012,564	2,824,308	6.36
unLSP GH4169	28,366	35,842	47,167	37,125	25.50
μLSPwC GH4169	1,071,356	1,248,742	1,589,690	1,303,263	20.21

## Data Availability

The original contributions presented in this study are included in the article. Further inquiries can be directed to the corresponding author.

## Abbreviations

The following abbreviations are used in this manuscript:

SLM	Selective laser melting
TRS	Tensile residual stress
HT	Heat treatment
μLSPwC	Microscale laser shock peening without coating
17-4PH	AISI 630
TC4	Ti-6Al-4V
GH4169	Inconel 718
unLSP	Specimens not subjected to μLSPwC
CRS	Compressive residual stress
SP	Shot peening
LPB	Low plasticity burnishing
LSP	Laser shock peening
Nd:YAG	Neodymium-doped yttrium aluminum garnet
SEM	Scanning electron microscope
EBSD	Electron backscatter diffraction
CV	Coefficient of variation
FCI	Fatigue crack initiation
FCG	Fatigue crack growth
FFZ	Fatigue fracture zone
